# Mediating effect of moral sensitivity and professional identity between moral courage and compassion fatigue among nursing interns: a cross-sectional study

**DOI:** 10.1186/s12912-024-02173-8

**Published:** 2024-08-13

**Authors:** Lijuan Yi, Jian Cai, Ting Shuai, Maria F. Jiménez-Herrera, Lei Gu, Xu Tian

**Affiliations:** 1 Department of Nursing, Hunan Traditional Chinese Medical College, Zhuzhou, 412000 China; 2 Nursing Department, Universitat Rovira i Virgili, Tarragona, Spain; 3 School of Nursing, Yongzhou Vocational Technical College, Yongzhou, 425000 China; 4 Second Clinical Division, Peking University School and Hospital of Stomatology & Beijing Key Laboratory of Digital Stomatology, Beijing, 100081 China; 5 School of sports & arts, Hunan University of Chinese medicine, Changsha, 410000 China; 6 Chongqing Center for Evidence-based Traditional Chinese Medicine, Division of Science & Technology and Foreign Affairs the First Affiliated Hospital of Chongqing University of Chinese Medicine, No. 6 of 7th Brach of Panxi Road, Jiangbei District, Chongqing, 400020 China

**Keywords:** Nursing interns, Compassion fatigue, Moral courage, Moral sensitivity, Professional identity

## Abstract

**Background:**

Compassion fatigue in nursing interns contributes to career indecision and worsens the nursing shortage. While work environment and psychological factors are well-studied, the ethical dimension remains unexplored. Understanding these mechanisms, particularly the role of moral courage, is essential for designing interventions to combat compassion fatigue and address the workforce crisis. This study investigates the influence of moral courage on compassion fatigue among Chinese nursing interns, focusing on the mediating roles of moral sensitivity and professional identity.

**Methods:**

A quantitative, cross-sectional study was conducted in accordance with the STROBE guidelines. We used the convenience sampling method to recruit 467 nursing interns from four public junior colleges in Hunan Province, China in February, 2024. Data were collected using Compassion Fatigue Short Scale, Moral Courage Scale, Revised Moral Sensitivity Questionnaire, and Professional Identity Scale. Data analyses were conducted using SPSS 22.0 and Amos 21.0.

**Results:**

The modified model exhibited a good fit (χ^2^/df = 3.437, AGFI = 0.928, IFI = 0.984, TLI = 0.976, CFI = 0.984, NFI = 0.977, RMSEA = 0.072). Moral sensitivity positively influenced both moral courage and professional identity, while professional identity negatively impacted compassion fatigue. Importantly, the effect of moral courage on compassion fatigue was entirely mediated by moral sensitivity and professional identity (β = -0.114, *P* = 0.001).

**Conclusion:**

This study suggests that moral courage in nursing interns mitigates compassion fatigue through the combined mediating effects of moral sensitivity and professional identity. Ethics education programs fostering moral courage, moral sensitivity, and professional values in nursing students could be crucial in alleviating compassion fatigue.

## Introduction

Nursing is a profession built on compassion and a strong moral commitment. As student nurses transition into clinical internships, they encounter a unique set of challenges [1, 2] that include balancing academic pressures with the demands of patient care [3, 4], navigating complex healthcare environments, and providing emotional support to patients who are ill and vulnerable [3, 5–7]. These stressors contribute to compassion fatigue [8–10], a state of characterized by emotional exhaustion and secondary traumatic stress [11]. High rates of compassion fatigue among nursing students have been reported, negatively impacting their well-being, academic success, and the quality of healthcare services [12–20].

Existing strategies to address compassion fatigue are limited. However, internal resources such as moral courage, moral sensitivity, and professional identity can help individuals cope with stressful situations [21–24]. Moral courage is defined as the capacity and willingness to adhere to ethical standards and act according to personal ethical values despite potential negative outcomes or ethical dilemmas [25]. Research demonstrates that these resources can mitigate compassion fatigue across various caregiving professions [26–28], with moral courage playing a crucial role [26, 29]. Yet, the specific mechanisms by which moral courage influences compassion fatigue in nursing interns remain unclear.

Moral sensitivity, characterized by comprehending the ethical values associated with human health situations, is the ability to identify the presence of ethical issues [30–32]. For nursing interns, this capacity is crucial because it empowers them to make ethically-based decisions and take appropriate actions when confronted with ethical dilemmas [25, 33, 34]. This heightened moral sensitivity not only bolsters their courage to act in accordance with their beliefs but also fosters a deeper commitment to and greater hope in their chosen profession [35, 36]. Furthermore, a study by İlter et al. demonstrates that as the level of moral sensitivity increases, the degree of compassion fatigue experienced by intensive care nurses decreases [37]. However, whether this relationship holds true for nursing interns remains to be further investigated.

A strong sense of professional identity is critical for nursing students to develop into confident, successful, and resilient healthcare professionals [38, 39]. This identity encompasses both career planning process and the affirmation of their roles within their current clinical setting [40]. Research shows that nursing interns with a well-developed professional identity demonstrate a more positive attitude towards their future careers. This positive attitude not only fosters a deeper understanding of their professional value during internships but also leads to increased feelings of accomplishment and satisfaction, ultimately mitigating compassion fatigue [41, 42].

The cognitive-transactional model of stress by Lazarus and Folkman [43] offers a framework for understanding how individuals cope with stress, including in nursing and medical education [44, 45]. According to this model, individuals engage in primary and secondary appraisals when facing stress. Primary appraisal involving assessing the threat or challenge posed by the situation, while secondary appraisal involves evaluating coping resources [46]. Applying this model to nursing interns helps elucidate how moral courage, moral sensitivity, and professional identity interact to alleviate compassion fatigue.

During primary appraisal, moral courage can instill confidence and determination in nursing interns to take action when facing ethical challenges [47]. It can also enhance their moral sensitivity, as practical action makes them more adept at identifying and analyzing ethical issues [25, 48]. In the secondary appraisal, moral sensitivity enables healthcare providers to recognize and understand ethical issues, leading to more effective coping decisions in stressful situations [49, 50]. Some studies suggest that moral sensitivity is a prerequisite for moral courage; individuals must identify moral issues before taking action, indicating a complex interplay between the two [51]. Professional identity acts as an intrinsic resource throughout the appraisal and coping process. Evidence shows that moral courage can enhance the level of professional identity among nurses [52, 53]. Furthermore, a study conducted in China with 349 nurses found that moral sensitivity can directly enhance professional identity [54, 55]. Professional identity not only directly alleviates compassion fatigue but also provides more enduring psychological support by enhancing individuals’ professional self-esteem and job satisfaction [56, 57].

However, current research often focusing on one or two of these concepts in isolation [19, 26–28, 35, 37], with limited studies on nursing interns. This fragmented approach fails to reveal the interactions and combined effects of these variables. This study aims to investigate the impact of moral courage on compassion fatigue among nursing interns and explore the mediating roles of professional identity and moral sensitivity in this relationship.

Based on cognitive-transactional model and the literature review, we propose that moral courage enhances moral sensitivity, enabling nursing interns to better recognize and address ethical issues, thereby strengthening their professional identity. This interaction can further alleviate compassion fatigue. Therefore, professional identity and moral sensitivity may mediate the relationship between moral courage and compassion fatigue.

To address these aims, we proposed the following nine hypotheses:

### Hypothesis 1

Moral courage negatively influences compassion fatigue among nursing interns.

### Hypothesis 2

There is a positive reciprocal relationship between moral courage and moral sensitivity.

### Hypothesis 3

Moral courage positively influences professional identity.

### Hypothesis 4

Moral sensitivity positively influences professional identity.

### Hypothesis 5

Moral sensitivity negatively influences compassion fatigue among nursing interns.

### Hypothesis 6

Professional identity negatively inlfuences compassion fatigue among nursing interns.

### Hypothesis 7

Moral sensitivity mediates the relationship between moral courage and compassion fatigue.

### Hypothesis 8

Professional identity mediates the relationship between moral courage and compassion fatigue.

### Hypothesis 9

Professional identity and moral sensitivity serve as a chain mediating effect in the relationship between moral courage and compassion fatigue.

## Methods

### Study design

This quantitative, cross-sectional study employed a web-based questionnaire survey and adhered to the Strengthening the Reporting of Observational Studies in Epidemiology (STROBE) guidelines.

### Study setting and sampling

In a convenience sample from four public junior colleges in Hunan province, located in central China, eligible nursing interns were recruited in February, 2024. Inclusion criteria for participants were: [1] completion of at least two years of nursing coursework (including general nursing and midwifery specialties) [2], participation in clinical internships at second-level hospitals or above for a minimum of eight months, and [3] provision of informed consent and voluntary participation. Interns solely involved in clerical management or administration duties without direct patient contact were excluded.

### Sample size

This study employed structural equation modeling (SEM) with maximum likelihood estimation to examine all paths between variables. The N: q rule (10:1) was employed to determine the minimum sample size, where *N* represents the required number of cases and q signifies the number of parameters requiring estimation [58]. Based on this rule, with 28 estimated parameters (q), a minimum sample size of 280 was obtained. To account for a potential 20% invalid questionnaire rate, the target sample size was adjusted to 339. To ensure generalizability and minimize sampling bias, 544 nursing interns undergoing internships in public junior colleges were recruited.

### Data collection

An online questionnaire was created using the Wenjuanxing platform (https://www.wjx.cn). Nursing interns accessed the survey by scanning a QR code through their WeChat app. To ensure data quality, a pilot test was conducted beforehand to estimate completion time and inform necessary adjustments. System restrictions limited to one per device and WeChat account, and all questions were mandatory to guarantee complete data. Prior to the study, designated teaching counselors from four colleges received standardized training on research objectives and survey procedures. They then recruited eligible nursing interns who were fully informed about the study’s aims and methods and provided electronic written informed consent. To maintain the data integrity, responses completed in less than 8 min or exhibiting patterns of regularity, uniformity, or logical inconsistency were excluded. This rigorous process yielded a final sample of 467 valid responses from a total of enrollment of 544 nursing interns, resulting in a high effective response rate of 85.9%.

### Ethical consideration

This study was approved by the Medical Ethics Committee of Hunan Traditional Chinese Medical College (YXLL202401004). To ensure complete participants confidentiality, a fully anonymous questionnaire survey was conducted. All collected data were securely stored and accessed only by the research team for the sole purpose of this study.

### Instruments

#### Demographic characteristics

The demographic characteristics questionnaire was developed by the research team assessed variables including participants’ age, gender, field of specialization, hospital level of internship, motivations for choosing nursing, exposure to ethics coursework, participation in internship ethics training, future career aspirations, interest in the nursing profession, and self-reported health status in the past month.

#### Compassion fatigue short scale

The original Compassion Fatigue Short Scale (CFSS) developed by Adams et al. [59]. was translated and validated for the Chinese language by Sun et al [60]. This Chinese version retains the original two-dimensional structure, namely Burnout (BO) subscale (items 1, 2, 4, 6, 7, 9, 11, and 13) and Secondary Traumatic Stress (STS) subscale (items 3, 5, 8,10, and 12), for a total of 13 items. The Cronbach’s alpha coefficients demonstrated satisfactory internal consistency for the total scale (0.90) and both subscales (BO: 0.90, STS:0.80). Each item is rated on a 10-point Likert scale, ranging from 1 (“never”) to 10 (always), with a possible total score range of 13–130. Higher scores indicate greater severity of CF. In this study, the Cronbach’s α values obtained for the total scale 0.925, BO subscale (0.894), and STS subscale (0.897) demonstrated good internal consistency.

#### Moral sensitivity questionnaire-revised

The original Moral Sensitivity Questionnaire (MSQ), developed by Lützén et al. [21]. and subsequently revised in 2006 [36], is a tool used to assess moral sensitivity. The Chinese adaptation of the MSQ, translated and validated by Huang et al., consists of 9 items and 2 dimensions: moral responsibility and strength, as well as moral burden [61]. The Cronbach’s α coefficient for the total scale of MSQR was found to be 0.820, indicating good internal consistency. Each item is rated on a 6-point Likert scale ranging from “complete disagreement” [1] to “complete agreement” [6], resulting in a total score range of 9–54. A higher score indicates greater moral sensitivity. In this study, the total scale demonstrated excellent internal consistency with a Cronbach’s α coefficient of 0.890.

#### Moral courage scale

The original Moral Courage Scale (MCS) was developed by Numminen et al. [62], and the Chinese version was translated and validated by Wang et al [63]. The Chinese version of MCS comprises 21 items, which are categorized into 4 dimensions: moral integrity (item 1, 4, 9, 11, 12, 19, and 21), commitment to good care (item 5, 8, 14, 16, and 18), compassion and genuine presence (item 2, 10, 15, 17, 20), and moral responsibility (item 3, 6, 7, and 13). Cronbach’s α for the total scale was 0.905, indicating high internal consistency. Each item is assessed on a 5-point Likert scale ranging from “does not describe me at all” [1] to “describe me very well” [5], resulting in a total score range of 21–105. A higher score indicates greater levels of moral courage. In this study, the total scale demonstrated excellent internal consistency with a Cronbach’s α coefficient of 0.976.

#### Professional identity scale

The Chinese version of the Professional Identity Scale (PIS), which has been translated and validated by Lu et al., was employed in this study to measure professional identity [64]. PIS was initially developed by Brown et al. [65]. and demonstrated a Cronbach’s α coefficient of 0.71. PIS is a unidimensional scale comprising 10 items, with 5 items (items 1, 4, 5, 8, and 9) scored positively and the remaining 5 items (items 2, 3, 6, 7, and 10) scored negatively on a 5-point Likert scale ranging from “never” [1] to “always” [5]. A higher score reflects a stronger sense of professional identity. In this study, the Cronbach’s α coefficient for the scale was found to be satisfactory at an acceptable level of 0.816.

### Statistical analysis

In this study, descriptive statistics were first to summarize participant baseline characteristics and their scores in moral courage, professional identity, moral sensitivity, and compassion fatigue. Due to the large sample size, Pearson correlation analysis was employed to explore the interrelationships among these variables [66]. Bootstrap testing was then utilized to examine the mediating effects of moral courage and compassion fatigue. The fit of the structural equation model was assessed using the established criteria: chi-square to degrees of freedom ratio (χ^2^/df) < 3, comparative fit index (CFI) > 0.90, Tucker-Lewis index (TLI) > 0.90, and root mean square error of approximation (RMSEA) < 0.08 [67]. A two-tailed *p*-value of 0.05 was set for statistical significance. All statistical analyses were performed using IBM SPSS 20.0 and IBM Amos 21.0 software.

## Results

### Participant characteristics

Table 1 summarized the baseline characteristics of 467 nursing interns. The majority (56.1%, *n* = 262) were centered around the age of 20. Gender distribution skewed predominantly female, with 90.6% (*n* = 423) identifying as female. Nearly all participants (97.9%, *n* = 457) belong to the nursing specialization. Regarding motivation for choosing nursing, 42.4% (*n* = 198) were driven by personal interest, while 53.7% (*n* = 251) were influenced by friends and family. Allocation adjustment accounted for 3.9% (*n* = 18) entering the field. Over half of the students (53.7%, *n* = 251) hold a positive or very positive attitude towards the nursing profession. 88.7% (*n* = 414) currently intern at tertiary hospitals. Additionally, 92.5% (*n* = 432) have taken nursing ethics courses during their academic studies, while a smaller proportion (3.0%, *n* = 14) have studied medical ethics. During their internship period, 366 students received further training related to ethics. In terms of personal health assessment over the past month, nearly 61.9% of the students (*n* = 289) considered their health condition to be good, although 31.3% perceived their health as average. 72.2% of the participants (*n* = 337) expressed their intention to pursue a career in nursing post-graduation.

**Table 1:** Compassion fatigue among nursing interns with different sample characteristics (*n* = 467)

Characteristics	Categories	Frequency (%)	The score of compassion fatigue		
			Mean (SD)	t/F	*P*-value
Age	≤ 19 years	49 (10.5%)	41.69 (19.57)	2.31	0.076
	20 years	262 (56.1%)	34.61 (18.75)		
	21 years	125 (26.8%)	35.36 (18.58)		
	22 years≥	31 (6.6%)	39.45 (22.10)		
Gender	Female	423 (90.6%)	36.00 (18.92)	0.44	0.664
	Male	44(9.4%)	34.68 (0.95)		
Field of specialization	Nursing	457(97.9%)	35.73 (18.97)	-1.13	0.261
	Midwife	10(2.1%)	42.60 (24.70)		
Reasons for selecting the nursing field	Voluntary	198(42.4%)	30.90 (17.17)	12.71	**< 0.001**
	Influence of friends and family	251(53.7%)	39.25 (19.52)		
	Adjustment^1^	18(3.9%)	43.61 (21.489)		
The level of the hospital where the internship was conducted	Tertiary hospital	414(88.7%)	35.23 (18.74)	-2.04	**0.042**
	Secondary hospital	53(11.3%)	40.91 (21.25)		
Whether specialized ethics courses were offered during the academic period	Nursing Ethics	432(92.5%)	35.29 (18.64)	8.79	**< 0.001**
	Medical Ethics	14(3.0%)	29.71 (16.72)		
	Covered in other courses or not studied	21(4.5%)	52.10 (22.84)		
Whether ethical training was received during the internship.	Yes	366(78.4%)	40.40 (20.31)	2.70	**0.007**
	No	101(21.6%)	34.63 (18.59)		
Level of interest in the nursing profession	Extremely interested	36(7.7%)	24.22 (14.48)	22.62	**< 0.001**
	Interested	215(46.0%)	29.91 (16.32)		
	Neutral	183(39.2%)	42.77 (19.28)		
	Disinterested	28(6.0%)	51.21 (16.76)		
	Extremely disinterested	5(1.1%)	38.00 (25.71)		
Post-graduation employment directions	Continue working in the field of study	337(72.2%)	33.27 (17.86)	15.59	**< 0.001**
	Choose a different industry	49(10.5%)	48.24 (22.14)		
	Pursue further studies	81(17.3%)	39.25 (18.94)		
Health status in the past month	Good	289(61.9%)	30.67 (16.94)	35.45	**< 0.001**
	Average	146(31.3%)	42.74 (18.90)		
	Poor	32(6.9%)	51.53 (20.57)		

Note:^1^ in the context of enrollment or admission, this could imply being assigned to a different major than originally intended due to various factors such as enrollment quotas, test scores, etc.

### Scores of variables

Table 2 presents the scores of nursing interns on various psychosocial constructs, including compassion fatigue, professional identity, moral sensitivity, and moral courage. Compassion fatigue demonstrated an overall mean of 35.876 (SD = 19.103), with subscales for burnout (24.281, SD = 12.908) and secondary traumatic stress (11.595, SD = 7.944) contributing to this score. Nursing interns exhibited a strong professional identity, reflected in a high mean score of 37.610 (SD = 6.287). Moral sensitivity yielded a mean score of 37.000 (SD = 7.236), compromised of scores for sense of moral burden (15.073, SD = 3.718) and moral responsibility (21.927, SD = 4.403). Finally, moral courage displayed the highest overall mean score (69.647, SD = 16.057). Subscale scores within moral courage included moral integrity (23.323, SD = 5.453), commitment to quality patient care (16.242, SD = 3.986), compassion and true presence with patients (16.814, SD = 3.907), and moral responsibility (13.268, SD = 3.249).

**Table 2:** Correlations for variables measured in this study (*n* = 467)

Variable	Mean	SD	a	b	c	d	e	f	g	h	i	j	k	l
a. **PI**	37.610	6.287	1											
b. **CF**	35.876	19.103	− 0.527^**^	1										
c. **BO**	24.281	12.908	− 0.569^**^	0.950^**^	1									
d. **STS**	11.595	7.944	− 0.342^**^	0.861^**^	0.659^**^	1								
e. **MS**	37.000	7.236	0.319^**^	− 0.159^**^	− 0.191^**^	-0.073	1							
f. **MS1**	15.073	3.718	0.126^**^	-0.010	-0.054	0.064	0.870^**^	1						
g. **MS2**	21.927	4.403	0.418^**^	− 0.254^**^	− 0.268^**^	− 0.174^**^	0.909^**^	0.585^**^	1					
h. **MC**	69.647	16.057	0.230^**^	− 0.148^**^	− 0.157^**^	− 0.101^*^	0.487^**^	0.383^**^	0.476^**^	1				
i. **MC1**	23.323	5.453	0.233^**^	− 0.145^**^	− 0.151^**^	− 0.102^*^	0.481^**^	0.385^**^	0.466^**^	0.978^**^	1			
j. **MC2**	16.242	3.986	0.200^**^	− 0.136^**^	− 0.154^**^	-0.076	0.465^**^	0.382^**^	0.441^**^	0.968^**^	0.935^**^	1		
k. **MC3**	16.814	3.907	0.232^**^	− 0.145^**^	− 0.151^**^	− 0.104^*^	0.466^**^	0.351^**^	0.468^**^	0.961^**^	0.915^**^	0.898^**^	1	
l. **MC4**	13.268	3.249	0.222^**^	− 0.149^**^	− 0.153^**^	− 0.109^*^	0.466^**^	0.355^**^	0.467^**^	0.957^**^	0.908^**^	0.907^**^	0.908^**^	1

Note: PI, professional identify; CF, compassion fatigue; BO, burnout; STS, secondary traumatic stress; MS, moral sensitivity; MS1, sense of moral burden; MS2, moral responsibility and strength; MC, moral courage; MC1, moral integrity; MC2, commitment to good patient care; MC3, compassion and true presence with the patient; MC4, moral responsibility. ^*^Statistical significance at the level of 0.05 (two-tailed), ^**^Statistical significance at the level of 0.01 (two-tailed)

### Correlations for variables

Our findings demonstrated positive correlations between moral courage and both professional identity (*r* = 0.230, *p* < 0.01) and moral sensitivity (*r* = 0.487, *p* < 0.01). Conversely, moral courage exhibited a negative correlation with compassion fatigue (*r* = -0.148, *p* < 0.01). Interestingly, both moral sensitivity (*r* = -0.159, *p* < 0.01) and professional identity (*r* = -0.527, *p* < 0.01) were inversely associated with compassion fatigue. Additionally, a positive association was observed between moral sensitivity and professional identity (*r* = 0.319, *p* < 0.01). A detailed presentation of all correlations is provided in Table 2, except for the Professional Identity Scale.

### Structural equation modeling of the association of moral courage, moral sensitivity, professional identity, and compassion fatigue

**Fig. 1:**
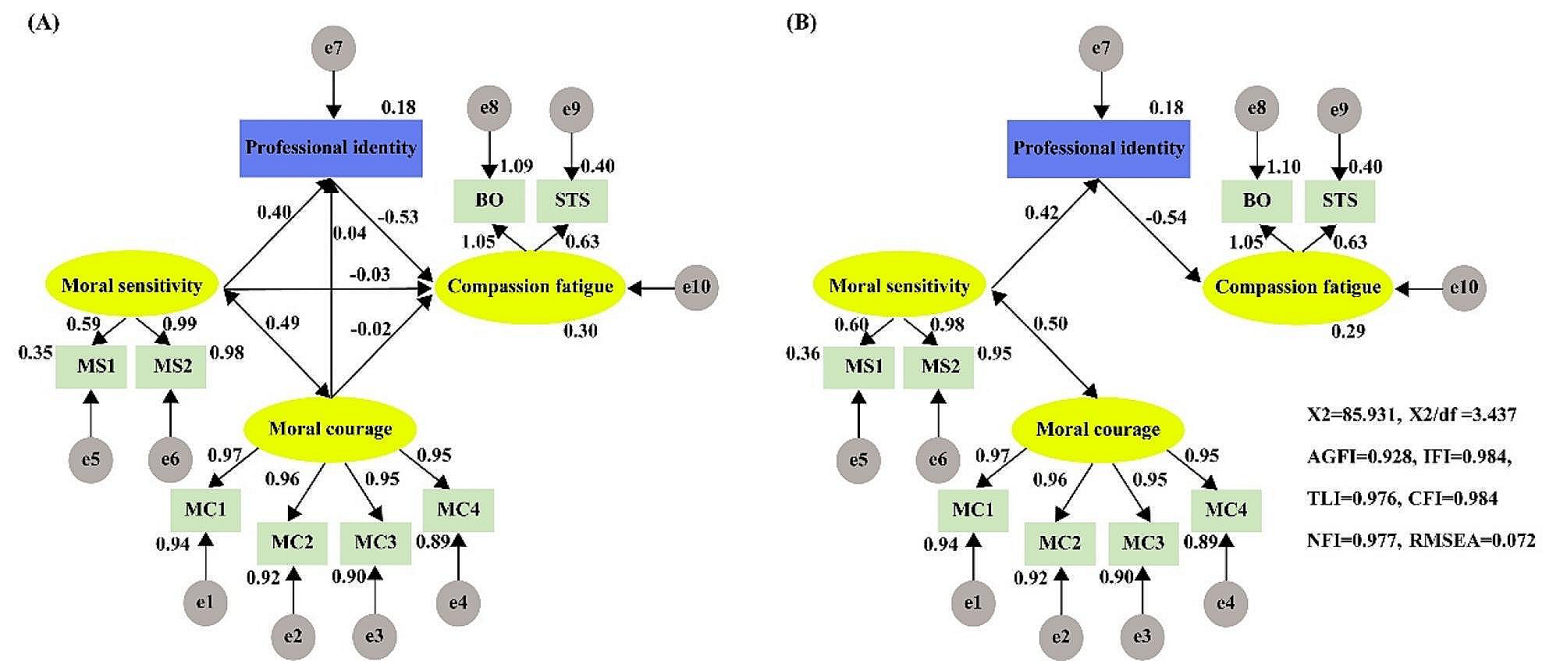
Theoretical mechanisms of moral courage on compassion fatigue according to correlation analysis (**A**), and influencing of moral courage on compassion fatigue through moral sensitivity and professional identity (**B**) Note: Abbreviations: MS1: moral responsibility and strength; MS2: moral burden; MC1: moral integrity; MC2: commitment to good care; MC3: compassion and genuine presence; MC4: moral responsibility; BO: burnout; STS: Secondary Traumatic Stress

Based on the results of the correlation matrix, we first established the relationship structure among all variables. Subsequent model fit analysis revealed that three direct paths were not statistically significant: moral courage to compassion fatigue, and moral sensitivity to compassion fatigue (Fig. 1A). To improve model fit, these non-significant paths were removed. The revised model demonstrated good fit indices: χ^2^/df = 3.437, AGFI = 0.928, IFI = 0.984, TLI = 0.976, CFI = 0.984, NFI = 0.977, RMSEA = 0.072 [90% CI, 0.056–0.089] (Fig. 1B).

As depicted in Fig. 1B, moral courage has a significant positive effect on moral sensitivity (β = 0.497, *p* = 0.001). Additionally, the direct paths from moral sensitivity to professional identity (β = 0.423, *p* = 0.001) and from professional identity to compassion fatigue (β = -0.543, *p* = 0.001) are statistically significant.

Table 3 summarizes the significance of all pathways obtained through a bootstrap test. The results reveal a statistically significant indirect effect of moral sensitivity on compassion fatigue mediated by professional identity (B = -0.230, 95% CI [-0.289 to -0.175], *p* = 0.001). Interestingly, moral courage has an indirect negative effect on professional identity mediated by moral sensitivity (B = 0.210 [95% CI (0.146 to 0.285), *p* = 0.001]. Furthermore, moral courage indirectly influences compassion fatigue through its combined effects on both moral sensitivity and professional identity (B = -0.114, 95% CI [-0.161 to -0.076]). These findings suggest a chain mediating role of moral sensitivity and professional identity in the relationship between moral courage and compassion fatigue among Chinese nursing interns.

**Table 3:** Scalar estimates of moral courage to compassion fatigue through moral sensitivity and professional identify

Pathway	Direct effect (95% CI)	Indirect effect (95% CI)	Total effect (95% CI)
Direct pathway			
Moral sensitivity ← Moral courage	0.497 (0.370, 0.607)	n.a	0.497 (0.370, 0.607)
Professional identify ← Moral sensitivity	0.423 (0.339, 0.503)	n.a	0.423 (0.339, 0.503)
Compassion fatigue ← Professional identify	-0.543 (-0.615, -0.456)	n.a	-0.543 (-0.615, -0.456)
Indirect pathway			
Compassion fatigue ← Moral sensitivity	n.a	-0.230 (-0.289, -0.175)	-0.230 (-0.289, -0.175)
Professional identify ← Moral courage	n.a	0.210 (0.146, 0.285)	0.210 (0.146, 0.285)
Compassion fatigue ← Moral courage	n.a	-0.114 (-0.161, -0.076)	-0.114 (-0.161, -0.076)

Note: CI, confidence interval; n.a., not available

## Discussion

Nursing interns are highly susceptible to compassion fatigue due to frequent exposure to others’ suffering, high-stress environments, and relentless self-sacrifice [68]. This fatigue can lead to increased dropout rates and early departure after graduation, ultimately worsening nursing staff shortages [12–19]. Therefore, understanding the mechanisms underlying compassion fatigue in nursing interns is essential for developing targeted and effective interventions.

Our study is the first, to our knowledge, to examine the mediating effects of moral sensitivity and professional identity on the relationship between moral courage and compassion fatigue among Chinese nursing interns. Interestingly, the average compassion fatigue score (35.876) measured by the CFSS was lower than reported in previous studies using the same tool [19, 69, 70]. This difference may be due to the timing of our research. Unlike prior studies conducted at the peak of the COVID-19 pandemic, ours was conducted after the situation had stabilized. Research suggests that healthcare professionals working in high-stress environments like pandemics experience more psychological distress [71, 72]. This can be attributed to frequent exposure to patient suffering and death [73]. The heightened risk of burnout and compassion fatigue among healthcare workers, including nursing students, during the COVID-19 pandemic is well-documented [74]. Therefore, the post-pandemic context of our study likely explains the discrepancy with previous findings.

Our study highlights the critical role of moral courage in enhancing moral sensitivity among nursing interns [53, 75–77], aligning with previous research on the positive correlation between these constructs in Chinese nursing interns [35, 78, 79]. The findings suggest that moral courage not only enhances nurses’ sensitivity to ethical challenges but also improves their capacity to navigate complex nursing environments by reinforcing their moral sensitivity [25, 80, 81]. This bidirectional relationship implies that while moral sensitivity lays the groundwork for the enactment of moral courage, moral courage subsequently strengthens this sensitivity [25, 80]. While Goktas et al. observed a slight negative correlation between moral courage and moral sensitivity among intensive care nurses [51]. The variance in findings may be attributed to differences in personality traits, such as individuals’ varying capacities to adapt and readiness to address moral challenges, as well as divergent internship and work conditions, including organizational support and the broader organizational climate. Additionally, our study found that the moral sensitivity and professional identity fully mediate the relationship between moral courage and compassion fatigue in the context of nursing education in China. These mediating roles are significant as they highlight the pathways through which moral courage can mitigate compassion fatigue. Specifically, enhancing moral sensitivity and professional identity in nursing interns can amplify the beneficial effects of moral courage on reducing compassion fatigue. This finding underscores the importance of developing and implementing training and intervention programs aimed at boosting these attributes. By doing so, we can not only enhance moral courage but also effectively alleviate compassion fatigue, supporting the ongoing professional growth of nursing interns. Interestingly, our study did not observe a direct effect of moral courage on professional identity or compassion fatigue, which diverges from existing research on nurses [26, 52]. This discrepancy may be attributed to differences in the study populations, the structural variations in compassion fatigue and professional identity measurement tools, or the specific healthcare environments in different international contexts. These findings highlight the need for further exploration into the relationship between moral courage and compassion fatigue among nursing interns, particularly focusing on the unique conditions and challenges faced by this group. Moral sensitivity, the ability to identify and address ethical dilemmas in nursing, is crucial for students’ professional growth [82]. It encompasses both empathy for patients’ needs [83] and a grasp of professional duties and ethics [84]. This study reveals a positive impact of moral sensitivity on professional identity formation in nursing interns. As moral sensitivity strengthens, so does their recognition of professional values [85, 86]. This empowers them to navigate ethical challenges and prioritize safety, quality, and ethics in daily practice [87–89]. Nursing interns with higher moral sensitivity are more likely to implement core values like patient respect, high-quality care, and rights protection [90], deepening their understanding of the profession’s significance. This fosters professional identity and a sense of fulfillment from helping others [91–94]. Notably, our study, alongside research on oncology, intensive care, and midwifery [28, 95], did not find a direct correlation between moral sensitivity and compassion fatigue. This might be due to two factors. Firstly, as newcomers, interns face fewer clinical challenges, potentially lessening the impact on both moral sensitivity and compassion fatigue. Secondly, prior studies often relied on correlational analysis, which doesn’t establish causality or account for other influencing factors [96, 97]. Therefore, further research on this relationship among nursing interns is warranted.

A strong professional identity in nursing not only enhances students’ self-confidence and sense of belonging but also serves as a key factor in job satisfaction and career stability [98–101]. This study demonstrates a direct negative correlation between professional identity and compassion fatigue, aligning with existing literature. This phenomenon is likely attributed to the specific work challenges faced by nursing students during their internship. Nursing interns often experience a demanding workload, frequent department rotations, and pressure to adapt, which can challenge their sense of self-efficacy and professional identity [102, 103]. A reduced professional identity exacerbates interns’ perception of ethical dilemmas, which in turn intensifies feelings of helplessness and defeat at work, as well as the accumulation of negative emotions, such as fatigue, pain, anxiety, and depression, ultimately leading to compassion fatigue [104, 105]. In light of these findings, it is recommended that hospital administrators enhance the cultivation of professional identity among nursing interns by implementing personalized training and support programs, effectively mitigating compassion fatigue.

### Limitation

While this study provides valuable insights, its cross-sectional design limits our ability to establish causality between moral courage and compassion fatigue. Future research employing longitudinal designs could prospectively examine this mitigating effect. Additionally, the reliance on self-reported measures (e.g., MCS, CFSS) necessitates the use of a multiplicity of data collection methods in future studies to enhance data accuracy and reliability. Furthermore, we did not include the physical and mental health conditions of nursing interns as controlling variables, which could influence the level of compassion fatigue. Future studies should incorporate these variables for a more comprehensive understanding of the influences on compassion fatigue.

## Conclusion

This study initially reveals that moral courage is positively associated with both moral sensitivity and professional identity, which in turn is negatively correlated with compassion fatigue. Further analysis confirmed that moral courage significantly reduces compassion fatigue mediated by both moral sensitivity and professional identity. These findings suggest that developing training programs grounded in moral courage to enhance moral sensitivity and professional identity is not only feasible but could also be highly effective in alleviating compassion fatigue among nursing interns.

## Data Availability

In consideration of the participants’ privacy within this study, the datasets generated and/or analyzed are not publicly accessible. However, they can be obtained from the corresponding author upon a reasonable request.
